# Health care as a universal right

**DOI:** 10.1007/s10389-016-0762-3

**Published:** 2016-08-09

**Authors:** Rui Nunes, Sofia B. Nunes, Guilhermina Rego

**Affiliations:** Faculty of Medicine of the University of Porto, Estrada da Circunvalação 9925, 4250-150 Porto, EU Portugal

**Keywords:** Accountability for reasonableness, Priorities in health care, Rationing, Right to health care

## Abstract

**Purpose:**

Most developed societies recognise the existence of a basic right of access to health care of appropriate quality, considering it a positive welfare right. It can even be one of the most important achievements of pluralistic and secular societies. The main objective of this study is to suggest the foundations for a universal right to health care, meaning the right of access to health care of appropriate quality. A second objective is to propose the necessary tools so that access to health care is viable in a specific commonwealth in accordance with available resources.

**Methods:**

To find this balance between an existing variable geometry and the actual level of resources of each specific commonwealth, the authors suggest the compatibility between Norman Daniels’ “accountability for reasonableness” and the integrated view of health of the World Health Organisation through the “equal opportunity function”.

**Results:**

The equal opportunity function appears to be an ethically acceptable solution for the existing variable geometry because it allows for different levels of provision and promotes an ethical rationing fully respecting accountability for reasonableness.

**Conclusion:**

The basic right of access to health care of appropriate quality is a fundamental humanitarian principle that should be enjoyed by all citizens of all countries, and the international community should recognise the obligation to promote these ideals by any means available. Indeed, although social rights such as health care demand citizens’ solidarity to be enjoyed, only with the universalisation of social rights will humanity be more equal in the future.

## Introduction

Most developed societies recognise the existence of a basic right to health care access, considering it a positive welfare right (Daniels 1998). It can even be one of the most important achievements of plural and secular societies—even a civilisation-based right—being considered as an expression of human dignity. In Europe, Art. 3 of the Convention on Human Rights and Biomedicine (Council of Europe 1996) implicitly recognised the existence of a right to health care access, even if limited by existent economic constraints. This article states that “Parties, taking into account health needs and available resource, shall take appropriate measures with a view to providing, within their jurisdiction, equitable access to health care of appropriate quality”.

The right to health care access is crucial to the pursuit of an effective equality of opportunities in a free and inclusive society. Diseases, deficiencies and disabilities, by restricting the opportunities that otherwise would be within the reach of the individual, should be regarded as unfair situations and not just the result of random forces of nature. That is, all citizens should have the necessary resources for an acceptable physical and psychological performance, thus being enabled to access a reasonable and appropriate range of social goods (Daniels 1985).

In the western world, one of the visions of distributive justice that is more in accordance with the conceptual formulation of the welfare state is perhaps the egalitarian theory of John Rawls, which rests on the concept of social contract (Rawls 1971). On the basis of this contract, a democratic and plural society, well organised and well structured, has as fundamental values individual freedom and equal access to primary social goods. The principle of equal opportunities becomes, therefore, the main instrument that determines the social, educational and health policies in the Western world. The existence of institutions legitimised by democratic power emanates from this model of social organisation, being, even for Rawls, a prerequisite for the widespread implementation of these values.

This means that the right to health care access should be interpreted in the light of egalitarian theories—namely, the precept of fair equality of opportunity. That is, all citizens should start their social lives with similar circumstances, on biological and social levels, so they can develop their talents and capabilities in accordance with the principle of individual freedom. However, utilitarian values should also be considered, such as the necessary cost containment in health care and cost-benefit, cost-utility and cost-effectiveness analysis.

The main objective of this study is to suggest a conceptual foundation for a universal right to health care access, meaning that all humankind should be enabled to access to health care of appropriate quality. This universal right is a moral right that could also become a legal one. A second objective is to propose the necessary tools so that access to health care of appropriate quality is viable in a specific commonwealth in accordance with available resources. With this architecture of principles, it is not intended to suggest that everyone in the world is entitled to every health service available or that everyone is entitled to the same health status because this status depends on many different circumstances, such as the familiar, social and economic conjuncture at that specific person and that specific community. Moreover, the authors did not intend to suggest a framework to prioritise health care access over other important goods, although a similar reasoning could be applied with this goal.

## The ethical background of a universal right to health care access

Health care access might be considered as a right of individuals and communities that should be implemented by the joint responsibility of citizens and society. Each state should promote and ensure access for all citizens to health care within the limits of the human, technical and financial resources (Abel-Smith 1994). However, health care necessarily has to compete with other social goods, so the only logical and consistent conclusion is that if we want a health system that effectively guarantees the access of all people, the resources should be used in treatments with proven effectiveness and with the least possible waste. That is, the implementation of a universal right to health care access should be based on a set of structuring principles:

1. Equal opportunity
2. Solidarity
3. Evidence-based practice

Thus, the first principle refers to the need to ensure equal access to health care of appropriate quality, overcoming at least some of the existing financial and non-financial barriers (Daniels and Sabin 2002). A concrete application of the principle of equal opportunities is the ideal that no citizen should be excluded from the health system because of lack of resources. Indeed, most developed societies claim the premise that all citizens should have access to the health system. To achieve a balance between the right of access to health care and the shortage of resources, it seems to be essential to define “appropriate quality,” which, in turn, will politically condition the constitution of a basic package of health care. The reasonableness criterion must preponderate, i.e., what the “reasonable” citizen would choose, given the circumstances (Williams et al. 2012).

The second principle aims to ensure that the way in which the basic ring of health care services is financed (in a tired system) ensures the principle of equity in access. In the perspective of equal opportunities, the economic and financial instruments that ensure the fulfilment of this principle are not particularly relevant. It is even irrelevant whether the financing of the health system is essentially based on taxes, as in the UK (Beveridge-type systems), on a compulsory public insurance according to the income of the citizens, such as in The Netherlands or Germany (Bismarck-type systems), or on both schemes, such as in mixed systems. What is at stake is, on one hand, equal opportunities in access to health care of acceptable quality and, on the other, promoting geographical equity. However, over the past decades, there has been controversy over the strengths and weaknesses of both Beveridge-type public integrated systems when compared to the Bismarckian type; each system must be adapted to the specificities of a particular culture (Veeder and Peebles-Wilkins 2001). However, an equitable funding presupposes some form of social solidarity. “Solidarity is the awareness of unity and a willingness to bear the consequences of it. Unity indicates the presence of a group of people with a common history and common convictions and ideals” (Choices in Health Care 1992). Solidarity can be voluntary, as when people behave out of humanistic motives, or compulsory, as when the government taxes the population to provide services to all.

In fact, although the financing of public health systems is already clearly progressive—through direct and indirect taxes charged to taxpayers—another issue relates to the possibility of introducing co-payments for services (consultation, surgery or diagnosing). However, the co-payment may have two distinct purposes: on one hand, disciplining and rationalising the demand for health care and, on the other, truly financing the system. However, it should be noted that, although there is no objection to the implementation of the principle of co-payments, the tax burden is relatively high in many countries. So, it may be socially unjust to double citizens’ taxes (co-payment and taxes), and this measure might jeopardise the social perception of the need for a public health system.

The problem of Welfare State financing is one of the most burning issues of controversy, because it is a serious matter with a direct influence on the quality of life of all people. Introducing the discussion around financing models—that is, if from the traditional model of tax-based financing we should evolve into a dynamic of user/payer—could give the impression that the only problem of the welfare state is the shortage of financial resources (Wall and Owen 1999). In short, accepting the principle of progressive taxation as one of the core criteria for the funding of the health system, and as a means to achieve fairness, it is imperative that there are politically established limits, these limits being imposed for two reasons: first, interpreting the principle established by Arthur Laffer in 1980, which reaffirms the conviction that there are direct and indirect tax evasions as well as other tax liabilities if the tax limits are unreasonable. An excessive progressivity will lead to a perverse and counterproductive effect because taxes would be perceived by the taxpayer as exaggerated. The second is according to the difference principle of John Rawls as this author argues that the existence of private property (substantially affected if taxes are exaggerated) is an essential tool to generate wealth in a fair and democratic society and thus achieve the ultimate goal of protecting the poorer strata of society.

The following criterion refers to a clear determination of the effectiveness of most diagnosis and treatment modalities in health, i.e. the acceptance of scientific evidence as an operational criterion. Evidence-Based Medicine (EBM) is the paradigm of this quiet revolution in clinical practice. The most common definition of EBM is taken from David Sackett: EBM is “the conscientious, explicit and judicious use of current best evidence in making decisions about the care of the individual patient. It means integrating individual clinical expertise with the best available external clinical evidence from systematic research” (Sackett 2000). This concept should be seen as a valuable tool for physicians and patients. The higher the level of evidence is, the greater the degree of clinical recommendation will be.

This definition is part of the dynamics of the doctor-patient relationship, which is in constant search of the best interest of the patient and his or her quality of life by enrolling in a dynamic perspective of medical practice (Leathard 2000). In fact, doctors and patients have never had access to so much information with regard to health care, through various means at their disposal. Unfortunately, much of this information is confusing or is biased and fragmented. For the clinician, it can be particularly difficult to discern which information is based on the latest scientific evidence. EBM aims to provide the best evidence for physicians and patients to jointly adopt the best course of action.

To achieve this goal it is necessary to perform critical evaluations of evidence that originate systematic reviews of existing literature and that, along with local circumstances, originate practical guidelines. These clinical guidelines should always be integrated with individual patient information and can only be considered as mandatory exceptionally, since their compulsory character can distort the essence of the doctor/patient relationship; they should rather help clinicians make the most efficient and effective decision. Furthermore, the emergence of this new field of health intervention implies that Evidence-Based Medicine is also used as a tool for resource allocation. That is, the economic and financial system constraints and the objective application of distributive justice criteria require that the scarce resources allocated to health are used in clinical treatments with proven effectiveness.

These critical reviews of the evidence take the form of meta-analyses and mega-meta-analyses, in which specialised centres, coordinated at the global level (of which the Cochrane Collaboration Centre is a good example), statistically evaluate the results of several published studies on specific topics, thus extracting what seems to be the best possible evidence on the effectiveness of a particular treatment or intervention. Currently, this methodology is mostly being used for medications but could be applied to any health field.

If a treatment has no proven clinical effectiveness it is not ethically legitimate to use it as there is not a valid reason to include it in the basic health care provision [29]. Evidence-Based Medicine thus has another aspect: to allow the prioritisation of health care on the basis of effectiveness. However, the implementation of criteria for clinical effectiveness does not object in principle to the restriction of innovative and expensive treatments provided that this decision is transparent and shared with society through democratic and consensual procedures. Evidence-Based Medicine then has a double objective: to assist clinical practice and to restrict treatments with unproven effectiveness according to criteria of distributive justice. It is increasingly seen as an essential tool for the provision of a reasonable health care level as well as to ensure fair access to health care (Wiseman et al. 2003).

However, equal opportunity, solidarity and evidence-based practice should be in accordance with an efficient use of resources and this should be a structural pillar of health policy. In the health area, economic rationality should have as its main objective to guarantee the right of access to health care of appropriate quality. Thus, the logic of the market and free competition in health must have as a prerequisite this basic right of the citizens. However, it is true that after a certain time, efficiency and equal opportunities play an opposite role, and from there the political decision must balance the interests at stake and decide according to the prevailing social values. For example, with waiting lists being structural to the health care system, which, by definition, is an open one, it is not possible to limit the demand for health care, and improving the efficiency and combating waste may reduce the waiting time to a socially acceptable minimum. However, from a certain point of view (where waste is null), pursuing economic efficiency might eliminate some types of treatment from the health system (Kapiriri et al. 2007).

It can be concluded that efficiency in resource allocation is an ethical imperative that society must fully assume. For example, the use of generic drugs (based on the assumption, not always valid, that this type of medicine has the lowest price on the market), safeguarding the principle of freedom of prescription and not jeopardising the best interests of the patient should be considered not an option but a professional duty. The presumable savings with the use of generics can channel resources to other areas of health (e.g. orphan drugs) and thus improve the overall performance.

Based on these criteria the authors suggest the existence of a universal right to health care as a basic right of humankind. By universal it is meant that everyone in every country should have access to health care of appropriate quality. However the concept of “appropriate quality” is context-specific because the world does not develop uniformly. Adjusting the right to health care access to the available resources (determined by each country’s political process) is the challenge suggested in this article.

## Setting limits to a universal right to health care access

Within the framework of the principles highlighted above, the existence of a universal right to health care access of appropriate quality is based on the premise that any person in any commonwealth is entitled to this moral right, although its specific legal operationalisation might be different in societies with different levels of development. On the other hand it is important to determine how to promote this right at a global level taking into consideration the absence of true universal enforcement institutions and of global governance arrangements (Brock 2009).

The existence of this universal right is part of a global perspective of justice, as suggested by Amartya Sen, in the sense of a global social choice (Sen 2009) emphasising that the principle of equal opportunities must be of transnational application ensuring the harmonious development of all people and all communities (Sen 1989, 1999). Indeed, in the absence of a global sovereign state/global sovereign institutions, what sort of international reforms can be implemented to make the world less unjust? John Rawls (1971), among other supporters of the “social contract” theory, relate distributive justice to the existence of sovereignty (Rawls 1971). In other words, they argue that only a sovereign state can apply a concept of justice by resorting to a perfect set of just institutions.

With this article, it is intended to claim that it is possible to reconcile the right to self-determination with the ideal of a “minimal humanitarianism” while there is not a perfectly fair world society. This “minimal humanitarianism” applies to a set of social goods—such as education, shelter, food and so on—but there is no doubt that health is amongst the most fundamental ones. Therefore, it is possible to consider the right to health care access as a basic and universal right, even if distinct communities have different rates of development and diverse availability of resources. This reality should not prevent the international community from establishing as an ideal a “spatial equity” meaning that any person in the world should have access to health care of appropriate quality.

Indeed, a thorough analysis of the Human Development Index clearly shows the variable geometry of development of different countries and communities (Human Development Report 2013). Variable geometry describes the idea of a method of differentiated integration of countries worldwide, which acknowledges that there are important, even irreconcilable, differences within the political, economic, social and cultural structures of different countries around the world; it therefore allows for different levels of access to health care and for different interpretations of “health care of appropriate quality”, but always in accordance with the United Nations concept of a decent standard of living.

For instance, recognising this variable geometry, the Treaty of Lisbon—a legal instrument that seeks to establish a letter of fundamental rights of all citizens of the European family—reflects the need to harmonise different cultures and social development models so that the collective future of the peoples of the European Union unfolds smoothly (Treaty of Lisbon 2007). This Treaty acknowledges in Article 168 that the “Union action shall respect the responsibilities of the Member States for the definition of their health policy and for the organisation and delivery of health services and medical care. The responsibilities of the Member States shall include the management of health services and medical care and the allocation of the resources assigned to them”.

Following this European experience a universal right to health care access of appropriate quality can be proposed worldwide if its limits are objectively accepted in accordance with the level of development of each specific country (Pogge 2008). The question to ask is, what medical care and health services should integrate the health care system? In this regard, Norman Daniels makes a clear distinction between preferences (amenities) and needs (fundamental) concerning health (Daniels et al. 1996). It is based on this distinction between preferences and needs that the famous Dutch report on health priorities (Choices in Health Care 1992) suggested the exclusion from the basic system of certain orthodontic treatments with a purely aesthetic purpose or certain psychotherapeutic interventions (counselling) that seek only to improve comfort and quality of life.

That is, based on the assumption that resources are limited and the cost of health care tends to grow exponentially, a fair society must establish, in accordance with predefined and mutually agreed rules, methods of inclusion and exclusion of certain basic package interventions (Gallego et al. 2011; Gibson et al. 2002). Indeed, rationing in health is due essentially to the increase in average life expectancy of the population, the increase in consumption of health care and of unprecedented development in medicine, namely the production of new pharmaceuticals, including pharmacogenomics (Teagarden et al. 2003). The authors agree with Norman Daniels and James Sabin (Daniels and Sabin 1997, 1998) in stating that as it is inevitable to set priorities in health care, rationing of scarce goods must be carried out in accordance with the principle of public accountability (Friedman 2008). That is, society should be informed about the criteria that guided these decisions by resorting to appropriate, fair and transparent procedures (Daniels et al. 2003). This implies the presence of a specific set of conditions, including the framework Daniels and Sabin designated as accountability for reasonableness: publicity condition, relevance condition, revision and appeals condition, and regulative condition.

To find the balance between an existing variable geometry and the actual level of resources of each specific commonwealth, a compatibility between Daniels’ “accountability for reasonableness” and the integrated view of health of the World Health Organisation is suggested. Indeed, the authors of this article propose an evolution of Daniels’ account of justice in health care access in which his “normal functioning” criterion as a standard for prioritising is changed to a more flexible, responsive and adjustable “needs criterion”. The perspective of justice as fairness implies that any citizen should have access to a decent minimum regarding health care, education and other social goods, guaranteeing therefore a decent standard of living.

**Fig. 1:**
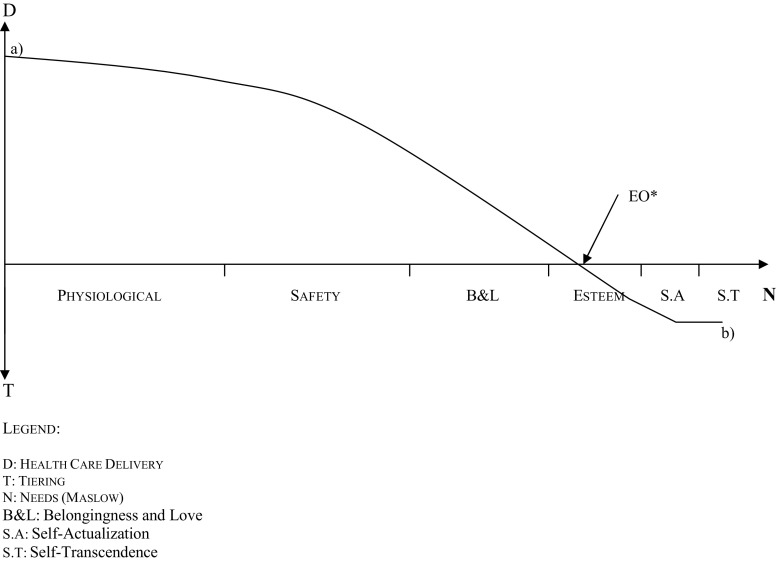
Equal opportunity function (EO)F. Legend: D: Health care delivery, T: tiering, N: needs (Maslow), B&L: belongingness and love, S.A: self-actualization, S.T: self-transcendence

However, the implementation of an effective opportunity for everyone should be re-evaluated in the light of available resources and the need to allocate them efficiently to different social goods. In a previous paper (2014) the authors suggested the implementation of a mathematical function that could be represented graphically in the form of an Equal Opportunity Function—(EO)F (Fig. 1). This function was suggested to promote an ethical reasoning that could support a framework for establishing priorities in health care within a specific community involving the convergence between the concepts of vertical and horizontal equity. Equal Opportunity Function suggests a theoretical approach that reconciles the way equals should be treated equally and unequal’s unequally. Two variables seem paramount for an accurate graphic representation of the (EO)F:

1. X-axis: represents the variable “hierarchy of needs”. The well-known Maslow needs are displayed in a progressive line: physiological, safety, belongingness and love, esteem, self-actualisation and self-transcendence needs;
2. Y-axis: Shapes the variable “type” and “level” of access to health care. The maximum value of this variable is represented by the universality and generality of the public services. The minimum value, below the X-axis, is left to the individual responsibility and not to the public beneficence.

In this perspective the deliverance of health care has two main components clearly separated by a specific moment—Point EO* at the Equal Opportunity Function. On the upper side of the Y-axis the financial burden of the system is publicly supported by the taxpayer. On the lower side of the Y-axis the mix between the “needs” criteria (that is, the lower level needs) and the financial constraints of the health care system implicate a personal, not social, level of commitment to one’s own health. Therefore, the public provision of health care is not mandatory anymore. As most health care systems in developed countries are segmented, alternative coverage schemes do exist, namely private health insurance or out-of-pocket payments. This Point EO* is variable depending of different variables and also on citizens’ democratic choices, but it can be situated schematically in the graphic area corresponding to what Maslow defined as esteem needs.

Ideally, with no resource constraints all citizens might have access to all health care services and to every kind of innovation in medicine. However, as stated by Penelope Mullen and Peter Spurgeon, “the demand for health care is infinite and so rationing is inevitable”, so the conceptual paradigm of the health policies worldwide is making explicit or implicit choices (Mullen and Spurgeon 2000). Indeed all societies face the problem of choice between competitive social goods and this trend is not slowing down. On the contrary, it is not possible, even in rich countries, to satisfy all the (Maslow) esteem and self-actualisation needs, nor all the individual preferences in health care. From an ethical perspective this framework allows conceptualising a fair way to establish priorities in health care and even to establish priorities between health care and other social goods.

The combination of Daniels’ “accountability for reasonableness”—namely the publicity, relevance, revision, appeals and regulative conditions—with the Equal Opportunity Function would allow determining, in a fair and transparent way, what the cut-off line between health care provision and personal responsibility should be in a specific commonwealth. The suggested framework could distinguish between high and low degree needs, for example a heart attack or high fever, although they need differential treatment. The Equal Opportunity Function would determine in advance if the treatment needed is or is not in the basic package, and afterwards the application of Daniels’ framework would validate this choice in a publicly accountable way. This methodology would also enable the decision-maker to choose between alternative treatments, although with very different costs, for instance, choosing which pacemaker to use in a specific circumstance, taking into consideration both the predicted clinical outcomes and the costs involved.

**Fig. 2:**
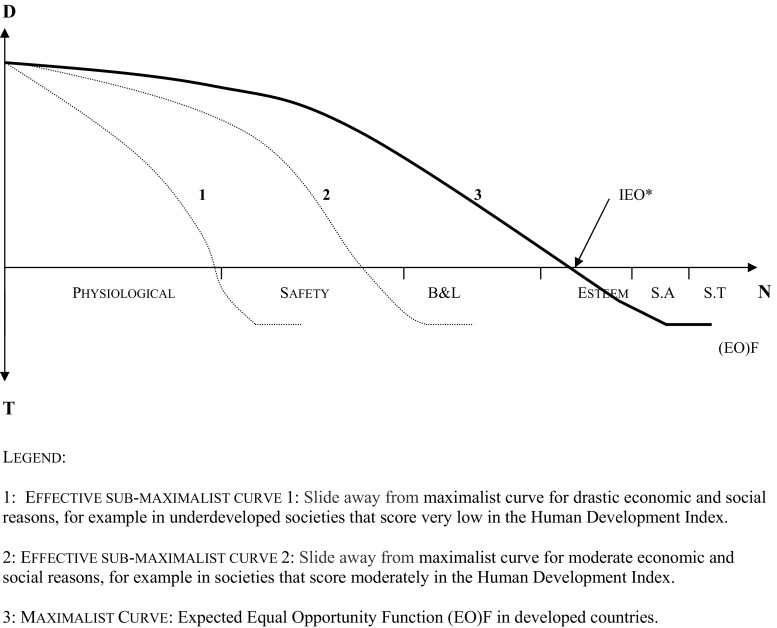
Equal Opportunity Function (EO)F: Variable geometry. 1: Effective sub-maximalist curve 1: Slide away from the maximalist curve for drastic economic and social reasons, for example in underdeveloped societies that score very low on the Human Development Index. 2: Effective sub-maximalist curve 2: Slide away from the maximalist curve for moderate economic and social reasons, for example in societies that score moderately on the Human Development Index. 3: Maximalist curve: Expected Equal Opportunity Function (EO)F in developed countries

Also this model is applicable in societies with very different levels of development in both the first and third worlds. And this perspective is also compatible with other axiological references such as the capacity to function or achieve a minimally decent life (Shue 1980; Held 1995; Nussbaum 2009; Venkatapuram 2011). The balance between the social goods that people may or may not be entitled to should preferably be reached through democratic arrangements, but always in accordance with a socioeconomically determined decision-making process. In less developed societies, and in direct accordance with the availability of resources, a progressive slide away from the maximalist curve might happen in accordance with the vertical equity principle (Fig. 2). Higher grade needs should always prevail over lower health care needs.

The concrete application of the equal opportunity function should take into consideration accountability for reasonableness (Hasman and Holm 2005; Jansson 2007; Rid 2009; Syrett 2008). This means that publicity, relevance, revision and appeals and regulative conditions should be applied by each commonwealth to determine the exact point EO* meaning the concrete level of health care available to each citizen. However, it should be emphasised that societal duties of health promotion are also underwritten by the principle of beneficence (Kelleher 2014). Both personal as well as public beneficence should be reflected in any society’s health policy and any political community is under a justice-based obligation of specific beneficence towards their members.

At a global level, if all human societies are regarded as a large political community, efforts should be made so that these values are universally shared and the correlative rights are also universally enjoyed. Also, the fact that hundreds of millions of people on this planet do not have access to health care of appropriate quality does not preclude the proposal of realistic ways of approaching this dramatic problem. The virtue of the Equal Opportunity Function strategy, namely the possibility that there is a progressive slide away of the curve adjusting the deliverance to the existing resources, is that it can be applied in any country, whatever the level of development, without interfering with the right of self-determination of that specific community. Indeed, it is the progressive implementation of a social structure that enables citizens to enjoy basic rights that could promote a different evolution in the world’s order in the future. And civilised countries should promote these values sustainably so that every country would be compelled to implement a public health care system, although with different rhythms.

With regards to underdeveloped countries this perspective could be the starting point for the construction of a public health care system that will steadily develop in accordance with available resources in a fair and publicly accountable way. Moreover, such a transparent system could be internationally monitored and therefore external resources could be more easily conveyed by the social responsibility of developed countries or even of transnational corporations.

This ethical framework suggests that the moral and legal concept of a universal right of access to health care of appropriate quality might be indexed to the level of the economic development of a particular community. Therefore, different degrees of health care provision are still compatible with a universal dimension of this right. It is true that the quality of the health systems in developed countries depends largely on their economic power, but this assumption does not preclude the possibility of a progressive implementation of a health care system in accordance with the financial constraints of that particular community. Moreover, the steady development of such a system might be an example of societal transformation well received by developed countries stimulating its willingness to contribute to such an endeavour.

One should bear in mind that it is not possible to change the world’s order unilaterally, nor is it possible to surpass the longstanding right of self-determination of all countries. Therefore, rich countries have an ethical obligation to distribute the vast resources at their disposal fairly in a pragmatic and realistic way. Although not ideal, this framework has at least the potential to provide health care of appropriate quality to all people worldwide. So, this approach, far from legitimating the existing inequalities at a worldwide level, appeals for a true world change in income distribution and the international community should be mobilised with regard to these ideals. Also, the international institutions should consider health care as other social rights, a global priority, and contribute by peaceful means to a sustainable development of all countries.

## Conclusions

The universal right of access to health care of appropriate quality should be considered as a civilisation-based right from both the individual perspective and a social point of view. A healthy society is a more cohesive, more balanced and more productive one. Just as in the last decades cultural and economic globalisation has resulted in a sustained increase in well-being levels on a global scale, so international efforts to implement human rights in a more accelerated manner should exist. If the rights of cultural minorities are nowadays on the global political agenda, social rights such as health care access should also be considered a priority, notwithstanding the fact that they are welfare rights that demand citizens’ solidarity to be enjoyed. But only with the universalisation of social rights will humanity be more equal in the future.

Although the global poverty rate declined considerably between 1990 (37.1) and 2015 (9.6) (The World Bank 2015), the contribution of the international community would still be an important step to overcome the global disparities between rich and poor countries and to solve many problems related to lack of food, education, shelter and health in these countries. Moreover, because this contribution is only residual it is frequently conveyed by NGOs and not by the states (Global impact 2016). Both an increase in those contributions and that countries’ specificities should be taken into consideration are naturally recommended so that the international financing of social goods is efficient and effective. Thus, specific cases of international policy should be directed to the needs of each country. Indeed, if food, education, shelter and health are considered as basic human rights, this means that they are universal, inalienable and interdependent and should be internationally guaranteed (Office of the United Nations High Commissioner for Human Rights 2006).

However, all human rights impose different types of obligations on states, namely the need to take positive steps to their fulfilment. The proposed equal opportunity function appears to be an ethically and even politically acceptable solution for the existing variable geometry because it allows for different levels of provision of health care and promotes an ethical rationing fully respecting accountability for reasonableness. Also, this approach allows for different levels of priorities is health care that are context-dependent. For instance it is possible to prioritise health care in Norway or in South Africa with the equal opportunity function as an ethical background. Indeed, and for various reasons, despite a significant improvement of indicators of socio-economic development of the western democracies over the past decades, even developed countries need to implement some sort of framework to fairly establish priorities in health care.

It is beyond the scope of this article to determine how the universal right of access to health care of appropriate quality might be propelled in countries and communities that have other social needs beyond health care, but the power of international institutions and of a global ethical conscience might be a good starting point. Indeed, this basic right is a fundamental humanitarian value that should be enjoyed by all citizens of all countries and the members of the international community should recognise the obligation to promote these ideals by any available means.
